# How to Help Young Children Ask Better Questions?

**DOI:** 10.3389/fpsyg.2020.586819

**Published:** 2021-01-12

**Authors:** Azzurra Ruggeri, Caren M. Walker, Tania Lombrozo, Alison Gopnik

**Affiliations:** ^1^MPRG iSearch | Information Search, Ecological and Active Learning Research with Children, Max Planck Institute for Human Development, Berlin, Germany; ^2^TUM School of Education, Technical University of Munich, Munich, Germany; ^3^Department of Psychology, University of California, San Diego, San Diego, CA, United States; ^4^Department of Psychology, Princeton University, Princeton, NJ, United States; ^5^Department of Psychology, University of California, Berkeley, Berkeley, CA, United States

**Keywords:** question asking, information search, information gain, scaffolding, vocabulary, preschoolers

## Abstract

In this paper, we investigate the informativeness of 4- to 6-year-old (*N* = 125) children’s questions using a combined qualitative and quantitative approach. Children were presented with a hierarchical version of the 20-questions game, in which they were given an array of objects that could be organized into three category levels based on shared features. We then tested whether it is possible to scaffold children’s question-asking abilities without extensive training. In particular, we supported children’s categorization performance by providing the object-related features needed to ask effective constraint-seeking questions. We found that with both age and scaffolding children asked more effective questions, targeting higher category levels and therefore reaching the solution with fewer questions. We discuss the practical and theoretical implications of these results.

## Introduction

As language develops, children add *question asking* to their expanding active-learning toolbox, which already includes many other types of self-directed exploratory actions, such as looking, pointing, crawling, approaching and avoiding people, grabbing and manipulating objects. Question asking is a particularly powerful tool for improving the quality of social learning (Callanan and Oakes, 1992; Chouinard, 2007), as it allows young learners to be more precise about the information they want from social partners, select which informants to query, inquire about absent objects or events, address abstract concepts or emotions, and target specific attributes of the same object. Importantly, question asking also allows children to make queries targeting different levels of abstraction (e.g., “Do you like apples?” versus “Do you like fruits?”). Leveraging hierarchical structure in the world allows the learner to rule out multiple hypotheses at each step of the search process, making question asking more effective. For example, if you want to know what your child prefers for a snack, asking whether she wants fruit would probably eliminate many of the alternatives at once, and would be more effective then asking about each option one by one (“Do you want a banana? … maybe an apple? What about a cupcake? Some cookies?”).

In this paper, we go beyond previous work in two ways. First, we examine the informativeness of 4- to 6-year-old children’s (*N* = 125) questions using a combined qualitative and quantitative approach. Second, we investigate whether it is possible to scaffold children’s question-asking abilities by supporting their categorization performance.

## The Developmental Trajectory of Question Asking

Previous studies have examined the quality of children’s questions using variants of the 20-questions game, in which participants have to identify a target object or category of objects within a given set by asking as few yes-no questions as possible (e.g., Mosher and Hornsby, 1966; Ruggeri and Lombrozo, 2015; Ruggeri et al., 2016). Researchers traditionally measured the quality of children’s questions by classifying them as *constraint seeking* or *hypothesis scanning*, where the former target a feature shared by multiple objects (e.g., “Is it a fruit?”), whereas the latter target an individual object (e.g., “Is it the apple?”). When all candidate solutions are presented as equally likely to be correct, constraint-seeking questions are at least as informative as (and often much more informative than) hypothesis-scanning questions: By ruling out multiple hypotheses at each step of the search process, they reduce the number of questions needed to identify the solution (Mosher and Hornsby, 1966). Seminal work indicates that preschoolers almost always ask hypothesis-scanning questions, and still predominantly rely on this strategy until age 7 (Mosher and Hornsby, 1966; Herwig, 1982; Ruggeri and Feufel, 2015; Ruggeri and Lombrozo, 2015). For example, Herwig (1982) found that about 95% of the questions asked by preschoolers, 90% of those asked by first graders, and 83% of those asked by second graders were hypothesis scanning. Other developmental studies found that 80% of the questions asked by fifth graders were constraint seeking and that this strategy further increased in prevalence until adulthood (see Ruggeri and Feufel, 2015; Ruggeri and Lombrozo, 2015; Ruggeri et al., 2016).

However, hypothesis-scanning questions can be more informative than constraint-seeking questions when the hypotheses available are not all equally likely (Ruggeri and Lombrozo, 2015). Moreover, crucially, not all constraint-seeking questions are equally informative. In this sense, the *qualitative* distinction between constraint-seeking and hypothesis-scanning questions does not necessarily map onto their actual informativeness. Given this consideration, recent work has gone beyond examining the *types* of questions that children ask to quantitatively measuring the *informativeness* of their questions, by calculating their expected stepwise information gain (see Ruggeri et al., 2016). Information gain assesses how much each question reduces the entropy, that is, the uncertainty (Shannon, 1948) in the hypothesis space considered. This is a much more fine-grained measure, able to distinguish among constraint-seeking questions, to account for the specific characteristics of the problem space (e.g., the likelihood of the considered hypotheses), and to capture the quality of children’s and adults’ question-asking strategies in absolute terms.

Generally, studies employing quantitative measures of question informativeness have confirmed earlier qualitative findings, namely, that the informativeness of children’s question-asking strategies increases with age (Ruggeri and Feufel, 2015; Ruggeri and Lombrozo, 2015; Ruggeri et al., 2016). This research has also demonstrated that children are *ecological learners*, able to tailor the types of questions they ask or select to maximize informativeness in a particular task. For example, Ruggeri and Lombrozo (2015) showed that 7-year-olds are more likely to ask hypothesis-scanning questions when there is a high-probability hypothesis available for their question to target. However, they are more likely to ask constraint-seeking questions when all hypotheses are presented as equally likely. In fact, recent work shows that even 5-year-olds flexibly rely on different question types (Ruggeri et al., 2017) and exploratory actions (Ruggeri et al., 2019) depending on their actual informativeness in the given learning context.

## The Present Paper

In this paper we go beyond the prior work reviewed in the introduction to examine the early development of question-asking abilities. Specifically, we focus on the transition between children’s ability to *recognize* better constraint-seeking questions—which is in place by age 5 (Ruggeri et al., 2017)—and their ability to effectively *generate* them, which begins to be reliably observed by age 7 (Ruggeri and Lombrozo, 2015).

We ask two important questions within this critical developmental window. First, how does the ability to generate effective constraint-seeking questions develop over the preschool years? To answer this question, we use a hierarchical version of the 20-questions game, in which participants are given an array of 16 monsters and are tasked with finding out which *kind* of monsters turns on a music machine, by asking as few yes-no questions as possible. Crucially, monsters can be classified into a symmetrical nested structure, organized at three category levels based on shared features, each including the same number of objects. At the higher level, monsters can be distinguished by pattern (eight dotted and eight solid monsters), at the middle level by shape (e.g., among the dotted monsters, four are spiked and four are triangular), and at the lower level by color (e.g., among the triangular monsters, two are red and two are green). This structure allows us to examine the level of abstraction children target with their questions (e.g., higher, middle, or lower). That is, we can go beyond recording the qualitative distinction between hypothesis-scanning and contraint-seeking questions to consider differences in informativeness among constraint-seeking questions. We will also implement a formal analysis to calculate the expected stepwise information gain of each question asked (Ruggeri et al., 2016).

Second, we consider whether it is possible to help young children to ask better questions. Previous attempts to improve children’s question-asking strategies have achieved only moderate success, and presented contradicting evidence. For instance, Denney (1975) trained 6-, 8-, and 10-year-olds by providing them with explicit examples of adult models asking either hypothesis-scanning or constraint-seeking questions when playing a short 20-questions game. Six-year-olds’ question-asking strategy was unaffected at posttest, while 8-year-olds adjusted their strategy to imitate whichever adult model they observed. Ten-year-olds were unaffected by the hypothesis-scanning model, but increased their rate of constraint-seeking questions after having observed the constraint-seeking model. Despite this initial improvement, however, training benefits were no longer apparent one week later. In another study, Courage (1989) trained 4-, 5-, and 7-year-old children on a 20-questions game, with the experimenter providing explicit instructions about how to ask the more informative constraint-seeking questions. Only 5-year-olds showed significant improvements after this training (Courage, 1989). Along the same lines, more recent work shows that prompting children to provide explanations about carefully selected evidence observed in a training phase increased the informativeness of the questions 6- to 7-year-olds – but not 4- or 5-year-olds – generated at test (Ruggeri et al., 2019).

Developing an effective intervention is challenging because it is still unclear what drives the observed developmental differences in the effectiveness and flexibility of information search, that is, why it is so hard for younger children to generate good questions from scratch (see Jones et al., 2020). On the one hand, previous research has explained age-related changes in the effectiveness of children’s questions as the result of their increasing ability to represent and organize the space of hypotheses under consideration at different hierarchical levels. Indeed, Jones et al. (under review) provided the first evidence for a developmental change in children’s ability and propensity to infer and reason with more abstract hypothesis-space representations. On the other hand, younger children also have difficulties generating object-general features that can be used to cluster similar objects into categories (see Ruggeri and Feufel, 2015). Along these lines, Legare et al. (2013) found that the ability to identify and flexibly categorize objects based on alternative features (e.g., color and pattern) predicted how well preschoolers generated effective questions.

In this study, we aimed to improve preschoolers’ ability to generate effective questions without explicit training, by reducing categorization demands. If young children’s poor question-asking performance is mostly resulting from their limited ability to identify the categorical features to target with constraint-seeking questions, we should expect that providing these features would considerably boost performance. However, if their difficulties in generating questions from scratch have more cognitive roots, related, for example, to the ability to evaluate the relative informativeness of several given features, then such a simple intervention might not be enough to scaffold effective question asking. In the current study, we therefore test these two alternative claims by providing 4- to 6-year-olds with the features that could be used to categorize and target groups of objects at different hierarchical level with constraint-seeking questions.

## Materials and Methods

### Participants

Participants were 40 4-year-olds (18 female, *M* = 53.9 months; SD = 4.4 months; *n* = 22 in the No-Scaffolding condition), 41 5-year-olds (25 female, *M* = 65.1 months; SD = 3.3 months; *n* = 23 in the No-Scaffolding condition) and 44 6-year-olds (17 female, *M* = 77.2 months; SD = 3.8 months; *n* = 24 in the No-Scaffolding condition), recruited at local museums and schools in the Bay Area, California. IRB approval (protocol #2010–03-1013) was obtained and parents gave informed consent for their children to participate before the study. Twenty-seven additional children were excluded from analysis due to experimental error (i.e., the experimenter forgot to collect the tokens for the last two or three questions or made a mistake in delivering the instructions; *n* = 18) or technical failures (i.e., videos were not recorded or lost before we could code them; *n* = 9).

### Materials

A 20 cm × 15 cm × 15 cm cardboard box was used to build the music machine. The box had an 8 cm slot on top to insert cards, and contained a wireless doorbell that was not visible to the participant. A hidden remote switch, controlled by the experimenter, activated the doorbell, causing the machine to play a melody. Children were given 10 metallic washers (3 cm in diameter) to be used as tokens. Two 10 cm × 7 cm cards, one depicting a musical note and one depicting the same musical note with a red cross over it, were also used as memory aids.

#### Training Materials

**FIGURE 1:**
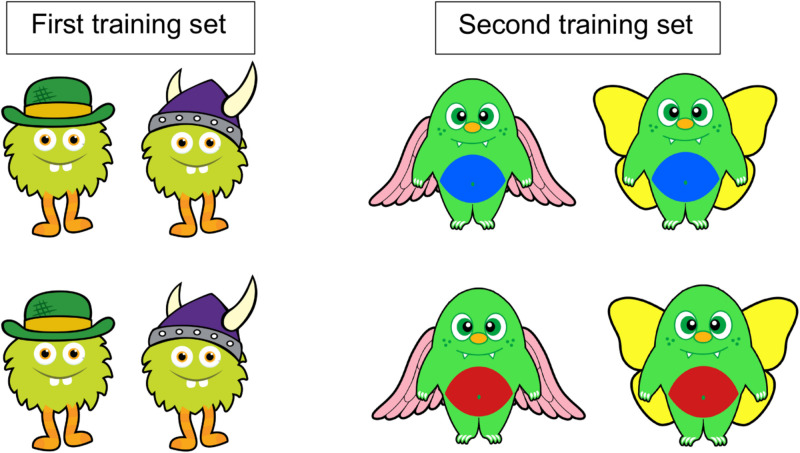
First **(left)** and second **(right)** training sets. The first set included four monsters, identical but for one feature—the hat. The second set included four monsters, identical but for two features—belly color and wings.

During training, eight 10 cm × 7 cm laminated cards were used, each depicting a colorful monster. The first training set (see Figure 1, left) included four monsters, which were identical but for one feature: Two monsters had a green bowler hat, whereas the other monsters had a purple Viking hat. The second training set (see Figure 1, right) included four new monsters, identical but for two features: belly color (two monsters had a blue belly, whereas the other monsters had a red belly) and wings (two monsters – one with a blue belly and one with a red belly – had pink wings, whereas the other monsters had yellow wings).

#### Testing Materials

**FIGURE 2:**
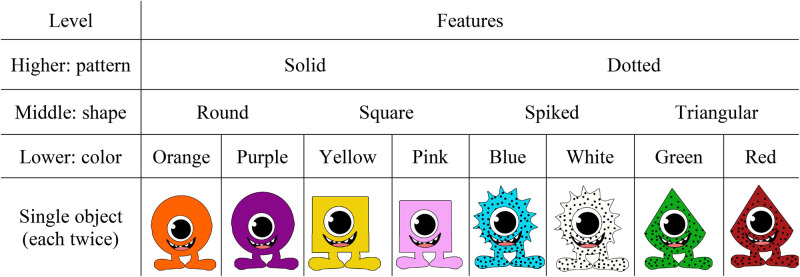
Set of cards used during the test phase, with an illustration of the hierarchical structure the monsters could be organized by based on their features.

At test, 16 10 cm × 7 cm laminated cards were used, each depicting a new monster (see Figure 2). Based on their body patterns (dotted versus solid), body shape (round, squared, spiked, or triangular) and body color (red, orange, yellow, green, blue, white, purple, and pink), the monsters could be organized in a hierarchical structure with two sets of monsters at the higher level (Pattern: eight solid and eight dotted monsters), four sets of monsters at the middle level (Shape: four round, four square, four spiked, and four triangular monsters), and eight sets for monsters at the lower level (Color: two orange, two purple, two yellow, two pink, two blue, two white, two green, and two red monsters).

### Procedure

The experimental session included a training and a test phase, and lasted about 10 to 15 minutes.

#### Training

The training phase was designed to familiarize children with the task and the stimuli. Children were presented with the music machine and shown that the machine plays music only when *some* cards are inserted into it. They were told that the cards represent the monsters, and that “When monsters jump in the machine, some of them turn it on. Other monsters don’t turn it on.” They were then presented with four cards representing the first training set of monsters (see Figure 1, right). The experimenter pointed out that each monster had a hat: Two of them had a green bowler hat, whereas the others had a purple Viking hat. The experimenter then told children that one type of hat makes the monsters turn on the machine, and that their task was to find out which kind of monsters turn on the machine by asking questions she can answer with “yes,” “no,” or “some.” Children were given an example: “You could ask if the monsters with the purple hat turn on the machine.” Children were prompted to ask that question, and were given a “no” feedback. They were then prompted to move the two monsters with the purple hat to the right side of the table, under the card depicting the crossed musical note (“no-music” pile), to help them remember that those monsters *do not* turn on the machine. Children were then prompted to generate a second question, this time by themselves. If a child failed to generate a question, the experimenter offered another example: “You could ask if the monsters with the green hat turn on the machine.” Children were prompted to ask that question, and were given a “yes” feedback. They were then prompted to move the monsters with the green hat to the left of the table, under the card depicting the musical note (“music” pile), to help them remember that those monsters *do* turn on the machine. Finally, children were prompted to insert the two monster cards from the “music” pile into the machine, to make it play music.

Next, children were presented with four cards representing the second training set of monsters (see Figure 1, right). This time, the experimenter pointed out that the monsters had different belly colors and different types of wings: Two of them had a blue belly, and two of them had a red belly; two of them had pink wings, and two of them had yellow wings. The experimenter then told the children: “It could be that one kind of belly makes the monsters turn on the machine, or it could be that one kind of wings makes the monsters turn on the machine,” and that their task was to find out which kind of monsters turn on the machine by asking questions about the monsters’ belly or wings, and that to those questions she could answer only “yes” or “no” or “some.” For this second training, children were prompted to generate questions by themselves. If they failed to do so, they were again offered examples: “You could ask if the monsters with the red belly turn on the machine” and “You could ask if the monsters with the yellow wings turn on the machine.” All questions generated were given “no” as feedback and moved under the “no-music” pile, until only one monster was left. The experimenter only answered “yes” to the last question, targeting the one monster left, that was moved to the “music” pile. Children were then prompted to insert the monster card from the “music” pile into the machine, to make it play music.

#### Test

At test, children were presented with 16 cards representing a new set of monsters (Figure 2), arranged on the table in a random fashion. Based on their pattern, shape, and color, the monsters could be organized in a hierarchical structure with a higher, middle, and lower level. Children were administrated the task in one of two experimental conditions: No-scaffolding or Scaffolding.

In the No-scaffolding condition, the experimenter said: “You have to find out which kind of monsters turns on the machine. To do this, you can ask me questions about the monsters. And to these questions, I can answer only ‘yes’, ‘no,’ or ‘some’.” In the Scaffolding condition, the experimenter added that the monsters have different body patterns, shapes, and colors: “It could be that the monsters with a specific pattern – dotted or not dotted – turn on the machine. Or, it could be that monsters with a specific body shape – square, round, spiked, or triangular – turn on the machine. Or, it could be that monsters of a specific color – orange, purple, white, pink, green, red, yellow, blue – turn on the machine.” The two conditions did not differ in any other way.

Children could ask as many questions as they wanted until they reached the solution, that is, until all monsters had been sorted into one of the two piles. However, to incentivize them to be efficient in their search, they were given 10 tokens, and were told they had to pay one token for each question they wanted to ask. All tokens children still had by the end of the game could be traded in for stickers. To ensure that the number of questions needed to reach the solution only reflected the informativeness of children’s question-asking strategies and was not affected by lucky guesses, children’s questions were always answered with a “no.” The solution to the game was always the last remaining hypothesis at the lower level (i.e., one pair of monsters of the same color). After each question, the experimenter prompted children to sort the targeted monsters into the “music” or “no-music” piles, as was done during training, and to then ask another question. Once all monsters had been sorted in one of the two piles, children were asked to explain why they thought only the two monsters in the “music” pile could turn the machine on (“Why do you think *these* two monsters can turn the machine on, while *those* ones cannot?”). Finally, children were prompted to insert both cards from the “music” pile into the machine to turn it on, and were given one sticker for each token they had left.

## Results

### Type of Children’s Questions

**FIGURE 3:**
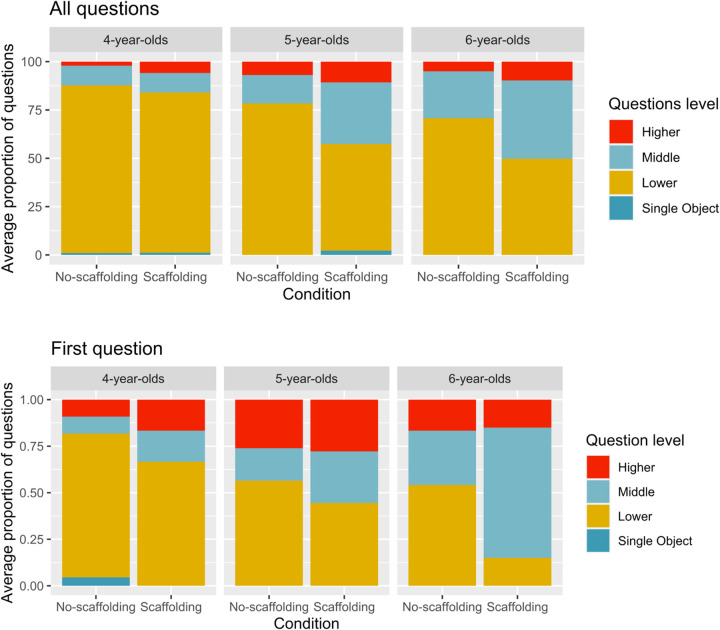
Average proportion of all questions **(top)** and first questions **(bottom)** asked that targeted higher-, middle-, lower-level categories and single objects, displayed by age, and condition.

Figure 3 (top) presents the average proportion of questions children asked that targeted higher, middle, and lower-level categories, as well as single objects. We analyzed these data using a series of univariate ANOVAs with the between-participants variables condition (Scaffolding and No-scaffolding) and age (4 vs. 5 vs. 6) as predictors. The complete set of analyses can be found in Supplementary Appendix A, together with a table with the number of questions asked per level that complements Figure 3. However, because these analyses are partially redundant, we focus here on the overall proportion of questions children asked that targeted the lower-level category, as it best captures the developmental and condition effect that can be observed in Figure 3.

This analysis revealed a main effect of condition, *F*(1,124) = 13.58, *p* < 0.001, η^2^ = 0.09 and age, *F*(1,124) = 11.42, *p* < 0.001, η^2^ = 0.16. Children in the Scaffolding condition asked fewer lower-level questions (*M* = 62%, SD = 27%) than children in the No-scaffolding condition (*M* = 78%, SD = 25%). Also, Bonferroni-corrected pair comparisons confirmed that 4-year-olds asked a higher proportion of lower-level questions (*M* = 85%, SD = 22%) than 5- (*M* = 68%, SD = 29%, *p* = 0.011) and 6-year-olds (*M* = 61%, SD = 26%, *p* < 0.001), with no difference between 5- and 6-year-olds (*p* = 0.620). No interaction was found (*p* = 0.144).

### Children’s First Questions

Figure 3 (bottom) presents the proportion of children targeting each level with their first query in the two conditions. As above, we focus here on the proportion of first questions that targeted the lower-level category. We conducted a logistic regression to analyze the impact of condition (Scaffolding and No-scaffolding) and age (4 vs. 5 vs. 6) on children’s likelihood of asking a first question targeting a lower-level category.

Anlayses revealed that children in the Scaffolding condition were less likely to ask first questions at the lower-level (41%), compared to children in the No-scaffolding condition [63%; *OR* = 2.83; (1.317, 6.282)], and that older children were less likely to ask first questions at the lower-level [Wald χ^2^ (2) = 13.8, *p* = 0.003]. Specifically, 6-year-olds were less likely to target the lower-level category with their first question (36%) than 5-year-olds [51%; *OR* = 0.17; (0.062, 0.445)], and 5-year-olds were less likely to do so than 4-year-olds [75%; *OR* = 0.32; (0.118, 0.841)]. Including an interaction term did not improve the fit of the model (*p* = 0.317).

### Mean Information Gain of Children’s Queries

**FIGURE 4:**
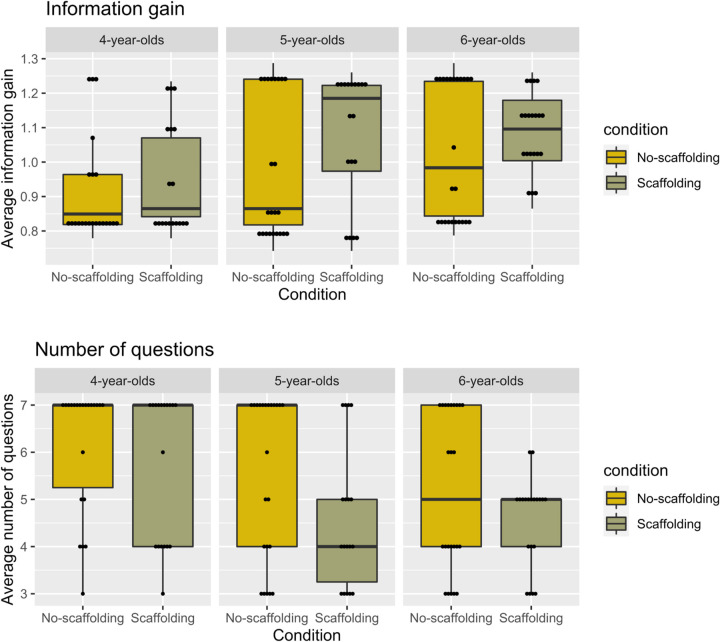
Average information gain **(top panel)** and number of questions **(bottom panel)** asked by children, displayed by age and condition. Dots represent individual participants’ scores; bold lines represent the medians.

Figure 4 (top) presents the average expected information gain of children’s queries. From a computational perspective, the hypothesis space in our game consists of 14 alternative hypotheses (*h*), corresponding to all of the categories at any hierarchical level. Matching the instructions given to participants, we assume a uniform prior. Because our task design allows for three possible answers to each question (yes/no/some), the information gain (IG) of each question is computed as:

$$\text{IG}=\log_{2}h-\left(\frac{h_{\text{yes}}}{h}\,\log_{2}h_{\text{yes}}+\frac{h_{\text{no}}}{h}\,\log_{2}h_{\text{no}}+\frac{h_{\text{some}}}{h}\,\log_{2}h_{\text{some}}\right)$$

where *h*_yes_/*h*_no_/*h*_some_ are the number of hypotheses that would remain under consideration, depending on the answer received. The optimal strategy consisted in targeting the higher level first, the middle level next, and the lower level last, reaching the solution with three questions, of average IG = 1.257.

An ANOVA with condition (Scaffolding and No-scaffolding) and age (4 vs. 5 vs. 6) as predictors revealed a significant effect of condition, *F*(1,124) = 4.10, *p* = 0.045, η^2^ = 0.03: Children asked more informative questions when scaffolding was provided. The analysis also revealed a main effect of age group, *F*(2,124) = 6.39, *p* = 0.002, η^2^ = 0.10. A Bonferroni *post hoc* analysis confirmed that the questions asked by 4-year-olds were less informative than those asked by 5- (*p* = 0.045) and 6-year-olds (*p* = 0.002), who did not differ from each other (*p* = 1.00). No interaction effect was found (*p* = 0.762).

### Number of Questions Asked

Figure 4 (bottom) presents the average number of questions children asked. A univariate ANOVA on the total number of questions needed to reach the solution confirmed the previous analyses, revealing a main effect of condition, *F*(1,124) = 6.07, *p* = 0.015, η^2^ = 0.05: Children required fewer questions to reach the solution when scaffolding was provided (see Figure 4). The analysis also showed a main effect of age, *F*(2,124) = 6.17, *p* = 0.003, η^2^ = 0.09. Bonferroni-corrected *post hoc* analyses found that 4-year-olds required more questions to reach the solution than 5- (*p* = 0.034) and 6-year-olds (*p* = 0.003), who did not differ (*p* = 1.000). No interactions were found (*p* = 0.790).

### Accuracy of Explanations

Children’s explanations for why the two monsters in the “music” pile activated the machine were transcribed and coded by two independent coders, one of them blind to the research hypotheses. Explanations were coded as ‘correct’ (1) if they referred to the feature differentiating those two monsters from the other monsters, i.e., their color (“Because they are both purple”), even when additional features were mentioned (e.g., shape and color; “They are purple and round”). Explanations were coded as ‘incorrect’ (0) otherwise. The two coders had 100% agreement.

Overall, 58% of the children provided a correct explanation. A logistic regression analysis indicated that condition (*p* = 0.003), but not age group (*p* = 0.082), made a significant contribution to prediction accuracy. Children in the Scaffolding condition had an increased likelihood of providing a correct explanation (73%) compared to children in the No-scaffolding condition [46%; OR = 1.178 (1.505, 7.012)]. Including an interaction term did not improve the fit of the model (*p* = 0.462). Notably, the percentage of children who were able to provide a correct explanation in the Scaffolding condition was well above chance performance (Binomial test: *p* < 0.001) – operationalized here as a random selection among the three related features (i.e., pattern, shape and color). Defining chance this way is quite conservative, since children were required to generate their explanations from scratch, and could have offered features that were entirely unrelated to the task (e.g., “these two monsters are cool”), provided redundant explanations (i.e., “because they can turn on the machine”), or said nothing at all – which they never did.

## Discussion

The presented findings demonstrate that providing children with features that can be used to generate constraint-seeking questions can substantially improve their search efficiency. In this sense, our results indicate that early difficulties with question asking are rooted in young children’s limited ability to identify categorical features that can be used to ask effective constraint-seeking questions.

In contrast with the mixed success of previous, more explicit attempts to improve the effectiveness of children’s spontaneous question asking (e.g., Courage, 1989), our findings demonstrate that it is possible to support children’s performance without extensive training. That said, effects of scaffolding manifested as a shift away from questions that targeted lower-level categories, rather than a shift towards the *optimal* approach (i.e., targeting higher-level categories). This pattern echoes recent results showing that prompting 6- and 7-year-olds to explain patterns of data supports a similar shift, away from lower levels of the hierarchy (Ruggeri et al., 2019). Thus, our results also suggest that younger children’s difficulties in generating questions from scratch might also be related to a fundamental cognitive limitation in their ability to appreciate and exploit the hierarchical structure of the hypothesis space, and therefore the relative informativeness of the available questions.

Our results also reveal a developmental increase in the effectiveness of children’s questions that was reflected in all measures: Compared to 4-year-old children, 5- and 6-year-olds asked more informative questions by targeting higher-level categories, allowing them to reach the solution with fewer questions. Note, however, that none of the analyses revealed an interaction effect, suggesting that the scaffolding was not more beneficial for one specific age group.

This study presents several limitations that should be discussed. First, we did not manipulate the features corresponding to the different levels of the hierarchy across participants (e.g., monsters at the lower level were always distinguished by color, at the middle level by shape, and at the higher level by pattern). However, a recent question-asking study with a similar set of stimuli and features showed no effect of the particular features assigned to the different category levels (e.g., whether color was assigned to the lower or to the higher level of the hierarchy did not have an impact on any of the considered question-asking measures; Swaboda et al., under review). We note, however, that because the stimuli were identical across conditions, this limitation is unlikely to have influenced the beneficial effect of the scaffolding provided. Second, we did not manipulate the order in which the experimenter presented the features in the Scaffolding condition. This limitation should be addressed in future work. Finally, it is unclear whether the effects of scaffolding that were found here would generalize to a new set of stimuli, or remain robust over time. Indeed, previous work shows that the beneficial effects of this type of interventions tend to be short-lived (see Denney, 1972). Moreover, this project, like most of the existing research on active learning, focused on identifying key developmental differences in the efficiency and adaptiveness of children’s search. We do not yet understand *why* these changes occur and what task-related (e.g., familiarity with the presented stimuli), social (e.g., parental input, socio-economic status), environmental (e.g., education), cultural and individual factors underlie the observed developmental trajectories. These represent important future directions, and necessary steps to bridge the gap with educational research.

Training children’s active inquiry skills at an early age has the potential to improve and accelerate the development of their general information search strategies and problem-solving skills, boosting their later and independent learning beyond the classroom. However, to develop robust, successful interventions, it is necessary to fully understand why previous attempts have (or have not) succeeded. We believe this requires that researchers first clarify the specific mechanisms underlying the developmental trajectory in children’s question-asking and, more generally, search strategies. The current study represents a first step toward a more formal approach to understanding and supporting active learning interventions for preschool-aged children.

## Data Availability Statement

The raw data supporting the conclusions of this article will be made available by the authors, without undue reservation.

## Ethics Statement

The studies involving human participants were reviewed and approved by Institutional Review Board University of California, Berkeley. Written informed consent to participate in this study was provided by the participants’ legal guardian/next of kin.

## Author Contributions

AR ideated and designed the studies, supervised data collection, analyzed the data, and wrote the first draft. CW contributed to the design, helped supervising data collection, and contributed to the manuscript. TL and AG supported the design and commented on the manuscript. All authors contributed to the article and approved the submitted version.

## Conflict of Interest

The authors declare that the research was conducted in the absence of any commercial or financial relationships that could be construed as a potential conflict of interest.
