# Supplementary material for: How to Help Young Children Ask Better Questions?
Source: Front Psychol. 2021 Jan 12;11:586819. doi: 10.3389/fpsyg.2020.586819 (PMC7874179; doi:10.3389/fpsyg.2020.586819)
Supplement: Supplementary file 1 [file Table_1.DOCX]

**Appendix**

**Appendix A**

**Type of children’s questions.** We first calculated the overall percentage of questions each child asked that targeted higher, middle, lower categories and single objects (see Figure 3, top, in the main text). We then analyzed this data using a series of ANOVAs with the between-participants variables condition and age as predictors. We found a main effect of condition on the percentage of higher- (*F*(1,124) = 4.81, *p* = .030, η^2^= .04), middle- (*F*(1,124) = 7.79, *p* = .006, η^2^= .05), and lower-level questions (*F*(1,124) = 13.58, *p* < .001, η^2^= .09), but not on the percentage of questions asked that targeted single objects (*p* = .336). Overall, children in the Scaffolding condition asked a greater number of higher- (*M* = 9%, *SD* = 11) and middle-level questions (*M* = 28%, *SD* = 25) than children in the No-scaffolding condition (Higher: *M* = 5%, *SD* = 10%; Middle: *M* = 17%, *SD* = 23%). Symmetrically, children in the Scaffolding condition asked fewer lower-level questions (*M* = 62%, *SD* = 27%) than children in the No-scaffolding condition (*M* = 78%, *SD* = 25%).

The analysis also revealed a main effect of age on the percentage of middle- (*F*(1,124) = 10.60, *p* < .001, η^2^= .15) and lower-level questions (*F*(1,124) = 11.42, *p* < .001, η^2^= .16), but no effect on the percentage of higher-level questions asked (*p* = .105) and on the percentage of questions targeting single objects (*p* = .434). Bonferroni-corrected pair comparisons showed that 4-year-olds asked a lower percentage of middle-level questions (*M* = 10%, *SD* = 19%) than 5- (*M* = 22%, *SD* = 24%; *p* = .058) and 6-year-olds (*M* = 32%, *SD* = 25%; *p* < .001), with no difference between 5- and 6-year-olds (*p* = .178). Also, 4-year-olds asked a higher percentage of lower-level questions (*M* = 85%, *SD* = 22%) than 5- (*M* = 68%, *SD* = 29%, *p* = .011) and 6-year-olds (*M* = 61%, *SD* = 26%, *p* < .001), with no difference between 5- and 6-year-olds (*p* = .620).

| Scaffolding condition | | | | | | |
| --- | --- | --- | --- | --- | --- | --- |
| Question level | 4-year-olds | | 5-year-olds | | 6-year-olds | |
| Higher | 5.83 | (9.74) | 10.74 | (11.32) | 9.72 | (12.14) |
| Middle | 10.12 | (18.83) | 31.85 | (23.65) | 40.54 | (22.67) |
| Lower | 82.93 | (22.88) | 55.18 | (27.66) | 49.75 | (18.31) |
| Single object | 1.11 | (4.71) | 2.22 | (9.43) | 0.00 | (0.00) |
| No-scaffolding condition | | | | | | |
| Question level | 4-year-olds | | 5-year-olds | | 6-year-olds | |
| Higher | 2.05 | (6.67) | 6.88 | (11.96) | 5.00 | (10.00) |
| Middle | 10.27 | (19.43) | 14.71 | (21.38) | 24.29 | (25.16) |
| Lower | 86.67 | (20.80) | 78.41 | (25.93) | 70.71 | (27.05) |
| Single objects | 1.01 | (4.71) | 0.00 | (0.00) | 0.00 | (0.00) |

Table A1. Mean percentage of questions (SD) asked by children that targeted higher-, middle-, lower-level categories and single objects, displayed by age and condition.
